# MHY1485 enhances X-irradiation-induced apoptosis and senescence in tumor cells

**DOI:** 10.1093/jrr/rrab057

**Published:** 2021-07-16

**Authors:** Lue Sun, Kumi Morikawa, Yu Sogo, Yuki Sugiura

**Affiliations:** Health and Medical Research Institute, Department of Life Science and Biotechnology, National Institute of Advanced Industrial Science and Technology (AIST), Central 6, 1-1-1 Higashi, Tsukuba, Ibaraki 305-8566, Japan; Health and Medical Research Institute, Department of Life Science and Biotechnology, National Institute of Advanced Industrial Science and Technology (AIST), Central 6, 1-1-1 Higashi, Tsukuba, Ibaraki 305-8566, Japan; Health and Medical Research Institute, Department of Life Science and Biotechnology, National Institute of Advanced Industrial Science and Technology (AIST), Central 6, 1-1-1 Higashi, Tsukuba, Ibaraki 305-8566, Japan; Health and Medical Research Institute, Department of Life Science and Biotechnology, National Institute of Advanced Industrial Science and Technology (AIST), 2217-14, Hayashi-cho, Takamatsu, Kagawa 761-0895, Japan

**Keywords:** mammalian target of rapamycin (mTOR), MHY1485, radiation, apoptosis, senescence

## Abstract

The mammalian target of rapamycin (mTOR) is a sensor of nutrient status and plays an important role in cell growth and metabolism. Although inhibition of mTOR signaling promotes tumor cell death and several mTOR inhibitors have been used clinically, recent reports have shown that co-treatment with MHY1485, an mTOR activator, enhances the anti-cancer effects of anti-PD-1 antibody and 5-fluorouracil. However, it remains unclear whether MHY1485 treatment alters the effects of radiation on tumor cells. In this study, the radiosensitizing effects of MHY1485 were investigated using murine CT26 and LLC cell lines. We examined mTOR signaling, tumor cell growth, colony formation, apoptosis, senescence, oxidative stress, p21 accumulation and endoplasmic reticulum (ER) stress levels in cells treated with MHY1485 and radiation, either alone or together. We found that MHY1485 treatment inhibited growth and colony formation in both cell lines under irradiation and no-irradiation conditions, results that were not fully consistent with MHY1485’s known role in activating mTOR signaling. Furthermore, we found that combined treatment with MHY1485 and radiation significantly increased apoptosis and senescence in tumor cells in association with oxidative stress, ER stress and p21 stabilization, compared to radiation treatment alone. Our results suggested that MHY1485 enhances the radiosensitivity of tumor cells by a mechanism that may differ from MHY1485’s role in mTOR activation.

## INTRODUCTION

Radiotherapy is a commonly used cancer treatment modality. Over 50% of cancer patients receive radiotherapy during the course of their disease [1]. While radiotherapy alone can cure early-stage tumors, it is usually combined with surgery or/and chemotherapy for the treatment of advanced tumors [1]. However, the recurrence of cancer from irradiated and adjacent areas, and the appearance of distant metastases is a serious problem, limiting the survival of patients [2–4]. Furthermore, radiation-induced side effects can limit the quality of life of patients [5]. Combining radiotherapy with molecular-targeting agents is expected to improve tumor control (local and systemic) and/or to decrease toxic effects on normal tissues [1].

Mammalian target of rapamycin (mTOR) is a serine/threonine kinase that is found in two discrete complexes, mTOR complex 1 (mTORC1) and mTORC2 [6]. It senses nutrient sources such as glucose and amino acids and plays a regulatory role in cell growth, metabolism and survival [7]. The mTOR pathways typically are upregulated in cancers (including gastric, prostate and breast) and have been shown to contribute to cancer progression and metastasis [8]. Several mTOR pathway inhibitors have been used in clinical settings, either alone or in combination with other therapeutic modalities [9]. Meanwhile, several studies have suggested that activation of mTOR signaling provides anti-cancer activity. Llanos *et al.* reported that mTORC1 stabilizes p21, and that protein expression of p21 and p-S6 (a surrogate for mTORC1 activity) correlates with improved survival in patients with head and neck cancers [10]. Kunisada *et al.* reported that metformin reduces tumor-infiltrating regulatory T cell numbers via activation of mTORC1 [11]. Furthermore, Chamoto *et al.* reported that co-treatment with MHY1485, an mTOR signaling activator, and anti-PD-1 antibody suppresses tumor growth more strongly than treatment with an anti-PD-1 antibody alone; this effect was mediated by activation of cytotoxic T cells [12]. Han *et al.* showed that MHY1485 treatment increases the 5-fluorouracil sensitivity of colon cancer cells deficient for p53 [13]. However, it remains unclear whether MHY1485 treatment alters the radiosensitivity of tumor cells.

In this article, we investigated the potential effect of MHY1485, alone and in combination with X-irradiation, using two tumor cell lines. We found that MHY1485 treatment inhibited growth and colony formation by both cell lines under irradiation and no-irradiation conditions, though the results were not fully consistent with MHY1485’s known role in mTOR signaling. Furthermore, we found that combined treatment with MHY1485 and radiation significantly increased apoptosis and senescence in tumor cells in association with oxidative stress, endoplasmic reticulum (ER) stress, and p21 stabilization, compared to the effects of radiation treatment alone.

## MATERIALS AND METHODS

### Cell line and culture conditions

Cells of the murine colon carcinoma cell line CT26 (harboring a *ras* mutation but wild type for *p53* [14]) and the Lewis lung carcinoma cell line LLC (harboring mutations in both *ras* and *p53* [15, 16]) were obtained from the American Type Culture Collection (ATCC; Manassas, Virginia, USA) and the Riken BioResource Center (Ibaraki, Japan), respectively. Both lines were cultured in RPMI1640 medium (Sigma-Aldrich Inc., Tokyo, Japan) containing 10% fetal bovine serum (FBS; Thermo Fisher Scientific, Tokyo, Japan), 100 mg/mL streptomycin and 100 U/mL penicillin (FUJIFILM Wako Pure Chemical Corporation, Osaka, Japan). Cells were incubated in a humidified atmosphere at 37°C with a 5% CO_2_. For subculturing, cells were rinsed with Ca^2 +^ − and Mg^2+^ − free phosphate-buffered saline (PBS; FUJIFILM Wako) and dispersed with 0.25% trypsin containing 0.5 mM ethylenediaminetetraacetate (FUJIFILM Wako). The number of cells were counted with a Countess II FL (Thermo Fisher Scientific) or a Scepter 2.0 (Merck KGaA, Darmstadt, Germany).

### Reagents, irradiation and experimental scheme

MHY1485 was obtained from Selleck Chemicals (Tokyo, Japan). For X-irradiation, a MBR-1520R-4 X-ray generator (Hitachi Power Solutions, Ibaraki, Japan) was used. Irradiation was performed using a tube voltage of 150 kV, a tube current of 20 mA, filters of 0.2 mm Cu and 0.5 mm Al and a dose rate of ~1 Gy/min.

The experimental scheme is shown in Supplementary Data 1. Plates were seeded with cells on Day 0. On Day 1 (after 24 h of culturing), cells were treated to MHY1485 or dimethyl sulfoxide (DMSO; vehicle control). On Day 2 (after 24 h of treatment), cells were subjected to 0 or 6 Gy X-irradiation. The cell numbers were counted daily on Days 3 to 5 to assess cell growth inhibition. The apoptotic and dead cell numbers, cell cycle, mitochondrial superoxide production, mitochondrial membrane potential, mitochondrial mass, lipid peroxidation and immunofluorescence were assayed on Day 3, and samples from Day-3 cultures were used for reverse transcription-polymerase chain reaction (RT-PCR) to assess gene expression. The levels of senescence-associated beta-galactosidase (SA-β-gal) were analyzed on Days 3 and 5. Colonies were fixed on Day 8 for use in the colony-formation assay.

### Cell growth inhibition assay

Cells were seeded in 6-well-plates (Corning, NY, USA) at 1 × 10^4^ cells/well and treated according to above scheme.

### Colony-formation assay

The colony-formation assay was performed as described previously [17, 18] Colonies were fixed and stained with methylene blue solution (0.25% methylene blue (FUJIFILM Wako) in 90% ethanol (FUJIFILM Wako). The number of surviving colonies that included 50 cells or more was counted. The surviving fraction was calculated based on the plating efficiency [19]. The sensitivity enhancement ratio at 6 Gy (SER) was calculated using the equation SER_(6)_ = log Sf_(MHY1485, 6)_ / log Sf_(DMSO, 6)_, where Sf_(MHY1485, 6)_ and Sf_(DMSO, 6)_ are the surviving fractions at 6 Gy following exposure to MHY1485 and DMSO, respectively [20].

### Apoptotic and dead cell analysis

Measurement of the ratios of apoptotic and dead cells was performed using the Annexin V-FITC Apoptosis Kit (BioVision, CA, USA) according to the manufacturer’s instructions. Briefly, cells were trypsinized and suspended in Binding Buffer. Annexin V-FITC and propidium iodide (PI) solutions then were added to the cell suspension. After a 15 min incubation at 4°C in the dark, cells were analyzed by flow cytometry using a BD Accuri C6 Plus (BD Biosciences, San Jose, CA, USA).

### Cell cycle analysis

Cells were trypsinized and fixed in cold (−30°C) 70% ethanol for two weeks. The fixed cells were washed in PBS and stained using the Cell Cycle Assay Solution Deep Red (Dojindo, Kumamoto, Japan) fluorescent probe. Cells were analyzed by flow cytometry using a FACS Canto II (BD Biosciences).

### Mitochondrial superoxide production, mitochondrial membrane potential, mitochondrial mass and lipid peroxidation analysis

Mitochondria-derived superoxide, mitochondrial membrane potential, mitochondrial mass and lipid peroxidation were detected using the MitoSOX Red (Thermo Fisher Scientific), JC-1 (Dojindo), MitoBright LT Green (Dojindo) and Liperfluo (Dojindo) fluorescent dye probes, respectively. Cells were trypsinized and incubated with MitoSOX for 10 min or with JC-1, MitoBright and Liperfluo for 30 min; fluorescence of the cells then was analyzed using a BD Accuri C6 Plus. The mean fluorescence intensity (MFI) for each sample was normalized to that for a control (0 Gy + DMSO) sample to calculate the relative fluorescence intensities for the samples [21].

### Measurement of senescence-associated β-galactosidase (SA-β-gal)

SA-β-gal levels were evaluated using the Cellular Senescence Detection Kit (Dojindo) according to the manufacturer’s instructions. Briefly, cells were trypsinized and suspended in RPMI1640. Cells then were incubated sequentially with bafilomycin A1 and SPiDER-βGal solution and MFI was obtained using a BD Accuri C6 Plus. The MFI for each sample was normalized to that for a control (0 Gy + DMSO) sample to calculate the relative fluorescence intensities for the samples [21].

### Immunofluorescence

Cells were trypsinized and fixed in 4% paraformaldehyde (FUJIFILM Wako) for 10 min. The fixed cells were washed and permeabilized in Permeabilization Wash Buffer (BioLegend, San Diego, CA, USA). Cells then were incubated sequentially with primary antibody (anti-phosphorylated (p)-mTOR [Ser-2448], anti-p-4E-BP1 [Thr37/46], anti-p-S6 ribosomal protein [Ser240/244], anti-p-Akt [Ser473], anti-p-SAPK/JNK [Thr183/Tyr185], anti-p21, anti-CHOP/GADD153, or anti-BiP/GRP78 antibody; Cell Signaling Technology, Danvers, MA, USA) and Alexa Fluor 488-conjugated secondary antibody (Abcam, Cambridge, UK). Cells were analyzed using a BD Accuri C6 Plus and MFI was obtained.

### Reverse transcription-polymerase chain reaction

Ribonucleic acid (RNA) isolation and subsequent reverse transcription were performed using the RNeasy Plus Mini Kit (QIAGEN, Tokyo, Japan) and High-Capacity cDNA Reverse Transcription Kit (Thermo Fisher Scientific), respectively. Quantitative real-time PCR was performed using PowerUp SYBR Green Master Mix (Thermo Fisher Scientific) and a CFX Connect Real-Time System (Bio-Rad, Hercules, CA, USA). Transcript levels were evaluated by the ΔΔCt method using the level of the beta-actin-encoding transcript as the control for normalization. Primer sequences were as follows: *beta-actin* forward: GATCTGGCACCACACCTTCT; *beta-actin* reverse: GGGGTGTTGAAGGTCTCAAA; *p21* forward: TGAGCCGCGACTGTGATG; *p21* reverse: GTCTCGGTGACAAAGTCGAAGTT.

### Statistical analysis

The mean and standard deviation (SD) were calculated for each quantitative parameter. The statistical analysis was performed using Prism 9 (GraphPad; San Diego, CA, USA). A two-tailed one-way analysis of variance (ANOVA) with a post hoc Dunnett’s multiple comparison test was used for comparing statistical differences in data from the cell growth inhibition assay. A two-tailed Student’s t test was used for comparing statistical differences in data from the colony-formation assay. A two-way ANOVA with a post hoc Sidak’s multiple comparisons test was used for comparing data from other assays. A p value less than 0.05 was considered statistically significant.

## RESULTS

### MHY1485 treatment inhibits tumor cell growth and surviving fraction

**Fig. 1.**
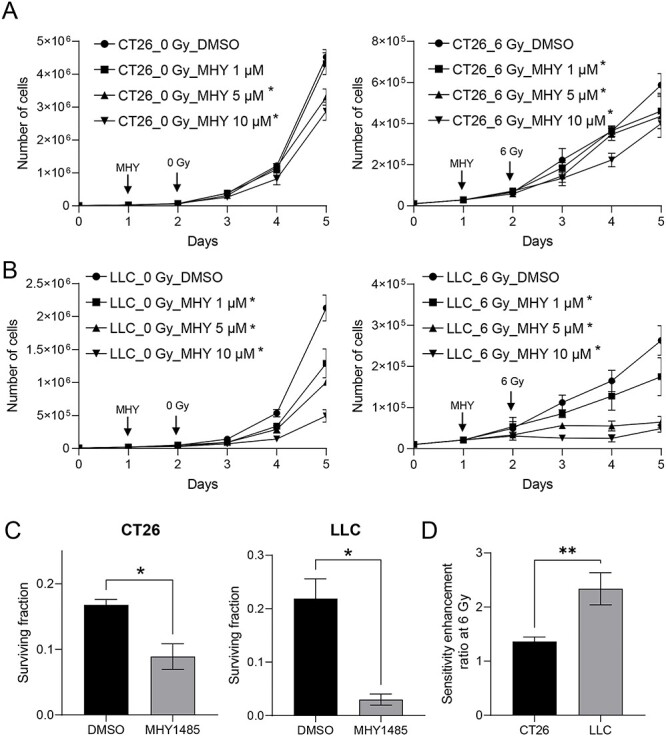
**MHY1485 treatment inhibits *in vitro* tumor cell growth and surviving fraction.** Tumor cell growth in (A) CT26 and (B) LLC. Addition of MHY1485 (MHY; at concentrations of 0-10 μM) and irradiation are indicated with vertical arrows. Cell numbers were determined once daily through Day 5 (i.e. 72 h after 0 or 6 Gy X-irradiation). All quantitative data are presented as mean ± SD (n = 4–5). ^*^ p < 0.05, two-tailed one-way ANOVA with post hoc Dunnett’s multiple comparisons test vs respective DMSO control. (C) Surviving fraction and (D) sensitivity enhancement ratio at 6 Gy in CT26 and LLC. (D) All quantitative data are presented as mean ± SD (n = 3). ^*^ p < 0.05, two-tailed Student’s t test.

First, we tested whether MHY1485 inhibited tumor cell growth. Figure 1A and B shows the changes in tumor cell number after treatment with MHY1485 and radiation (6 Gy), both alone and together. In CT26, treatment with 5 and 10 μM MHY1485 alone showed significantly delayed cell growth compared with no treatment; the combination treatment with 1, 5 and 10 μM MHY1485 and radiation showed significantly delayed cell growth compared with radiation alone (Fig. 1A). In LLC, treatment with MHY1485 at 1 μM or higher significantly delayed cell growth under both non-irradiation and irradiation conditions (Fig. 1B). These results suggested that MHY1485 suppresses tumor cell growth *in vitro*, whether administered alone or in combination with radiation. Interestingly, MHY1485 showed greater growth inhibitory effects with LLC than with CT26 (Fig. 1A-B), suggesting that LLC is more sensitive to MHY1485 than is CT26. Based on data reproducibility and a previous report [22], we selected 10 μM as the MHY1485 concentration for subsequent experiments.

We performed a colony-formation assay to investigate whether MHY1485 treatment enhances the radiosensitivity of tumor cells. In CT26, MHY1485 treatment alone resulted in a slight decrease in the colony formation rate (p < 0.05 as assessed by two-way ANOVA and post hoc Sidak’s test, but p > 0.05 as assessed by two-tailed Student’s t test), and co-treatment with MHY1485 and radiation decreased the colony formation rate compared to the that seen with radiation alone (p > 0.05 as assessed by two-way ANOVA and post hoc Sidak’s test; p < 0.05 as assessed by two-tailed Student’s t test) (Supplementary Data 2). In LLC, MHY1485 treatment alone decreased the colony formation rate (p < 0.05 as assessed by two-way ANOVA and post hoc Sidak’s test and by two-tailed Student’s t test), and co-treatment with MHY1485 and radiation decreased the colony formation rate compared to that seen with radiation alone (p > 0.05 as assessed by two-way ANOVA and post hoc Sidak’s test; p < 0.05 as assessed by two-tailed Student’s t test) (Supplementary Data 2). In both CT26 and LLC, the surviving fraction of cells following treatment with 10 μM MHY1485 was significantly decreased compared to that of DMSO-treated control cells after 6 Gy irradiation (as assessed by two-tailed Student’s t test) (Fig. 1C). These results suggested that MHY1485 has a radiosensitizing effect on tumor cells. In addition, LLC had a higher SER_(6)_ than did CT26, suggesting that MHY1485 is more effective at radiosensitizing LLC than CT26.

### MHY1485 treatment activates mTOR pathway under irradiation condition

**Fig. 2.**
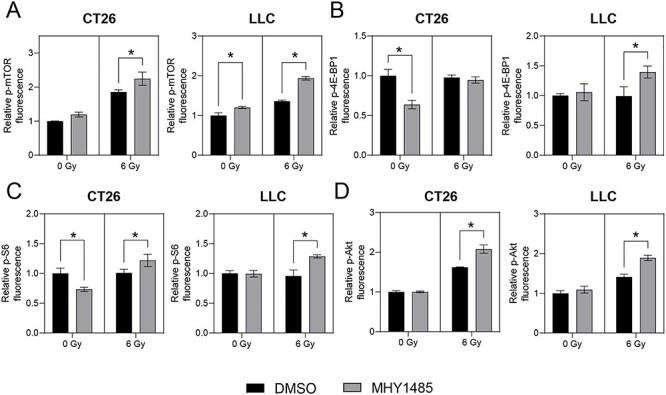
**MHY1485 treatment activates mTOR pathway in irradiated cell lines.** Immunofluorescence analysis of (A) phosphorylated (p) -mTOR, (B) p-4E-BP1, (C) p-S6 and (D) p-Akt protein. All quantitative data are presented as mean ± SD (n = 3). ^*^ p < 0.05, two-way ANOVA with post hoc Sidak’s multiple comparisons test vs respective DMSO control.

To analyze whether MHY1485 activates the mTOR pathway, we performed immunofluorescence analysis. Specifically, we used immunofluorescence to measure the intracellular levels of p-mTOR (mTOR phosphorylated at S2448, a molecule that is a biomarker of mTOR activation), p-4E-BP1 and p-S6 (phosphorylated versions of eukaryotic translation initiation factor 4E-binding protein 1 and ribosomal protein S6, respectively, molecules known to be downstream targets of mTORC1) and p-AKT (a phosphorylated version of protein kinase B, a molecule know to be a downstream target of mTORC2) [23, 24]. In CT26, treatment with MHY1485 alone did not result in significant accumulation of these phosphorylated proteins, while co-treatment with MHY1485 and radiation resulted in significant increases in the levels of p-mTOR, p-S6, and p-Akt (Fig. 2). In LLC, treatment with MHY1485 alone resulted in significant accumulation of p-mTOR only, while co-treatment with MHY1485 and radiation resulted in significant increases in the levels of p-mTOR, p-S6, p-4E-BP1 and p-Akt (Fig. 2). These results suggested that treatment with MHY1485 alone was not sufficient to activate all of the tested mTOR pathways under our experimental conditions, while the combination of irradiation and MHY1485 activate mTOR pathways. However, the net changes, even when significant, were less than 1.5-fold.

### MHY1485 treatment enhances apoptosis

**Fig. 3.**
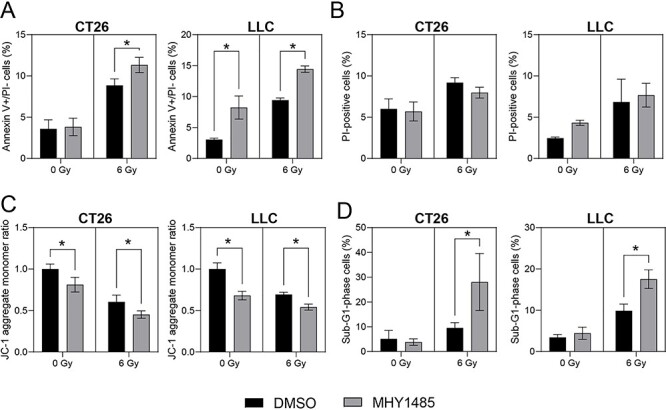
**MHY1485 treatment enhances apoptosis in cell lines.** (A) Proportion of early apoptosis (annexin V-positive and propidium iodide (PI) -negative) cells. (B) Proportion of dead (PI-positive) cells. (C) Relative mitochondrial membrane potential. (D) Proportion of early apoptosis (sub-G1-phase) cells. All quantitative data are presented as mean ± SD (n = 3–4). ^*^ p < 0.05, two-way ANOVA with post hoc Sidak’s multiple comparisons test vs respective DMSO control.

To investigate why MHY1485 inhibits tumor growth, we assessed apoptosis using flow cytometry. We employed a large gate in a Forward Scatter (FSC) vs Side Scatter (SSC) panel, with the intent of capturing all cells (Supplementary Data 3). We measured the proportions of early apoptotic cells and dead cells using the annexin V-FITC/PI double-staining method. In CT26, MHY1485 treatment alone did not increase early apoptosis (the proportion of annexin V- positive and PI-negative cells), while co-treatment with MHY1485 and radiation significantly increased early apoptosis compared to the level seen with radiation alone (Fig. 3A). In LLC, MHY1485 treatment significantly increased the level of early apoptosis under both non-irradiation and irradiation conditions (Fig. 3A). For both cell lines, MHY1485 treatment provided a non-significant change in dead (PI-positive) cells under both non-irradiation and irradiation conditions (Fig. 3B). Staining with the JC-1 dye revealed that the mitochondrial membrane potential was decreased for both cell lines in groups treated with MHY1485 (Fig. 3C). Furthermore, the proportion of the sub-G1 cells was increased in cells treated with the combination of MHY1485 and X-irradiation (Fig. 3D). Treatment with MHY1485 appeared to provide only limited effects on the cell cycle, but it should be noted that the histograms of the cell cycle in this experiment were broad (Supplementary Data 5). Together, these results suggested that MHY1485 enhances radiation-induced apoptosis of tumor cells.

### The combination of MHY1485 and radiation treatment enhances cellular senescence

**Fig. 4.**
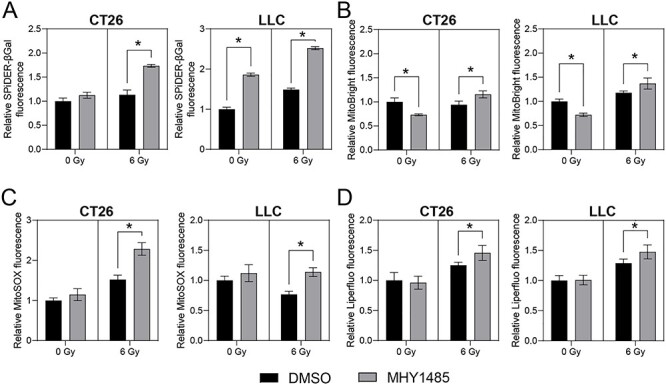
**MHY1485 treatment enhances senescence and oxidative stress.** Relative (A) senescence-associated β-galactosidase, (B) mitochondrial volume, (C) mitochondrial superoxide production and (D) lipid peroxide production. All quantitative data are presented as mean ± SD (n = 3–4). ^*^ p < 0.05, two-way ANOVA with post hoc Sidak’s multiple comparisons test vs respective DMSO control.

Tumor cell senescence also can be induced by radiotherapy or chemotherapy [25, 26]. We investigated senescence-associated phenotypes (increase of SA-β-gal, mitochondrial mass and oxidative stress) in MHY1485-treated tumor cells using flow cytometry. A small gate was used in FSC vs SSC to exclude annexin V- and PI-positive cells (Supplementary Data 3). First, we analyzed SA-β-gal levels on Day 3 (Fig. 4A) and Day 5 (Supplementary Data 4). Notably, similar results were obtained with samples from these two time points. For both CT26 and LLC, treatment significantly increased SA-β-gal levels under both non-irradiation and irradiation conditions, with the exception of the Day-3 results for CT26 under non-irradiation conditions (Fig. 4A and Supplementary Data 3). Given the similarity of results for Days 3 and 5, we chose to analyze mitochondrial mass and oxidative stress on Day 3. Interestingly, for both CT26 and LLC, MHY1485 treatment alone resulted in decreased mitochondrial mass, but co-treatment with MHY1485 and radiation resulted in significant increases in mitochondrial mass compared to that seen with radiation alone (Fig. 4B). To assess oxidative stress in treated cells, we measured the levels of mitochondrial superoxide and lipid peroxidation. For both CT26 and LLC, MHY1485 treatment alone provided limited changes in the levels of mitochondrial superoxide and lipid peroxidation, but co-treatment with MHY1485 and radiation resulted in significant increases in mitochondrial superoxide and lipid peroxidation compared to those seen with radiation alone (Fig. 4C and D). These results suggested that treatment with the combination of MHY1485 and radiation increases tumor cell senescence more than radiation alone.

### MHY1485 treatment increases the level of p21 protein, but not that of *p21* mRNA

**Fig. 5.**
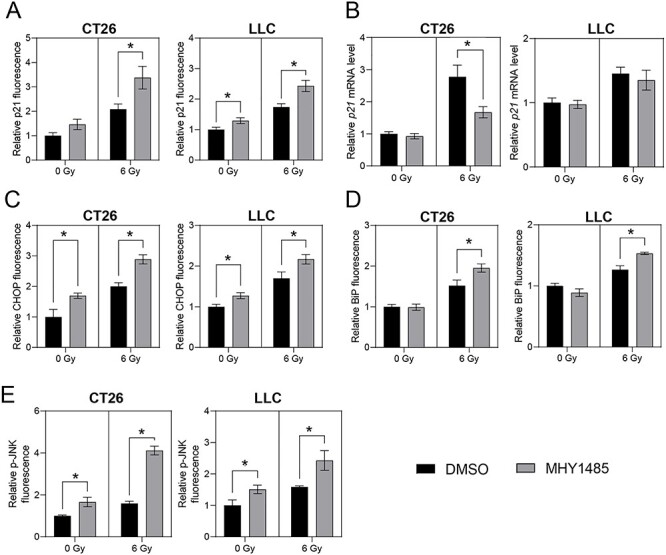
**MHY1485 treatment enhances p21 level and endoplasmic reticulum stress.** Relative levels of (A) p21 protein, (B) *p21* mRNA, (C) CHOP/GADD153 protein, (D) BiP/GRP78 protein and (E) p-JNK protein levels. All quantitative data are presented as mean ± SD (n = 3–4). ^*^ p < 0.05, two-way ANOVA and post hoc Sidak’s multiple comparisons test vs respective DMSO control.

Cell cycle arrest increases via p21 binding to, and inhibiting the activity of, cyclin-dependent kinase complexes [27], and p21 is also known as a marker of senescent cells [28, 29]. We assessed whether MHY1485 treatment affected the levels of p21 protein and mRNA. In CT26, treatment with MHY1485 alone provided a non-significant increase in the level of p21 protein, while co-treatment with MHY1485 and radiation resulted in a significant increase in the level of p21 protein compared to that seen with radiation alone (Fig. 5A). In LLC, MHY1485 treatment significantly increased p21 protein levels regardless of whether X-irradiation also was provided (Fig. 5A). These results suggested that MHY1485 treatment induces accumulation of p21 protein, which would in turn contribute to inhibition of tumor cell growth and increased senescence. In contrast, RT-PCR experiments revealed that, in both CT26 and LLC, MHY1485 treatment does not result in the accumulation of *p21* mRNA (Fig. 5B).

### MHY1485 treatment enhances ER stress

Although the ER stress response is a cytoprotective mechanism, overwhelming ER stress results in apoptosis [30, 31]. Furthermore, ER stress has been observed in senescent cells [32]. Therefore, markers of ER stress (specifically, levels of the C/EBP Homologous Protein [CHOP] and Binding immunoglobulin Protein [BiP]) [31] were analyzed in cells subjected to MHY1485 treatment. In both CT26 and LLC, MHY1485 treatment resulted in significant increases in the levels of CHOP protein, under both non-irradiation and irradiation conditions (Fig. 5C). For both cell lines, BiP levels were significantly increased in cells subjected to the combination of MHY1485 and radiation, compared to those in cells subjected to radiation alone (Fig. 5D). Furthermore, we analyzed levels of p-JNK (phosphorylated c-Jun N-Terminal Kinase), a mediator of CHOP expression and apoptosis [33]. In both CT26 and LLC, MHY1485 treatment resulted in significant increases in the level of p-JNK, under both non-irradiation and irradiation conditions (Fig. 5E). These results indicated that the apoptosis and senescence induced by co-treatment with radiation and MHY1485 may be associated with ER stress induction.

## DISCUSSION

It has been reported that MHY1485 activates mTORC1 and mTORC2 through direct binding to mTOR and/or activation of signaling upstream of mTOR in osteoblasts and hepatocytes [23, 34]. Notably, the activation of the mTOR pathways caused by MHY1485 treatment is counteracted by mTOR inhibitors (OSI-027, rapamycin and RAD001) [23, 34]. In our study, MHY1485 significantly increased the levels of phosphorylated mTOR and downstream targets under irradiation conditions, without an associated increase in the level of p-4E-BP1 in CT26 (Fig. 2). Although the observed changes in levels were small (1.5-fold or less) in these situations, the magnitudes of these changes were consistent with those reported in previous studies [34, 35]. In contrast, we found that treatment with MHY1485 alone (under non-irradiation conditions) provided limited increase in the levels of p-mTOR and downstream targets in CT26 and LLC (Fig. 2). These results were not consistent with the results of the growth inhibition and colony-formation assays, especially in LLC (Fig. 1), suggesting MHY1485 may not only activate mTOR, but also employ an antitumor effect mechanism of action distinct from that resulting in mTOR activation.

We found that MHY1485 treatment increased the proportion of annexin V-positive (early apoptosis) cells and senescence under non-irradiation conditions in LLC, although this effect was not seen in CT26 (Fig. 3A and Fig. 4). These results are largely consistent with the results for the growth inhibition and colony-formation assays. Furthermore, MHY1485 treatment increased apoptosis and senescence in both CT26 and LLC under irradiation conditions (Fig. 3A and Fig. 4). These results suggest that MHY1485 provides antitumor and radiosensitizing effects, at least in part through enhancement of apoptosis and senescence. ER stress and the associated unfolded protein response (UPR) have been shown to be induced by exposure to radiation [31], oxidative stress [36] and any of several types of chemical reagents [37]; similarly, overwhelming ER stress and UPR have been shown to result in apoptosis [30, 31] and senescence [38]. CHOP is a UPR-related protein that promotes apoptosis via activation of JNK signaling and proapoptotic proteins [39]. In the present study, we found that combination treatment with MHY1485 and radiation resulted in increased CHOP, BiP and p-JNK levels (Fig. 5C–E), suggesting that this co-treatment induces ER stress, which may induce apoptosis by activating the CHOP-JNK pathway. We also showed that the combination treatment with radiation and MHY1485 promotes senescence, with associated increase in p21 protein levels (Figs. 4 and 5A). However, *p21* mRNA levels were not increased under these conditions (Fig. 5B), suggesting that p21 protein is stabilized in co-treated cells. Previous studies have suggested that the acetylase activity of Tip60 is enhanced by ER stress [40] and that Tip60 may help stabilize p21 protein by suppressing proteasome-dependent degradation of p21 [41]; those results are consistent with those of the present study.

We note that our study did not address why MHY1485 treatment enhances ER stress. However, we found that treatment with the combination of radiation and MHY1485 increased mitochondrial volume, superoxide production and lipid peroxidation (Fig. 4B–D), suggesting that mitochondrial dysfunction and oxidative stress loading occurred in these cells. Mitochondria are considered the main source of reactive oxygen species (ROS), which are known to be triggers of apoptosis and senescence [42, 43]. Radiation exposure and ER stress loading has been reported to increase mitochondrial damage, mitochondrial volume and ROS levels [44–46]. MHY1485 also may enhance ER stress via inhibition of a late stage of autophagy [34]. Our results are consistent with these previous reports.

Our results consistently showed that LLC is more sensitive to MHY1485 than CT26. These two cell lines are derived from different strains and organs, and contain distinct genetic profiles. Interestingly, CT26 encodes wild-type p53, while LLC encodes a mutated version of this protein [14, 16]. Wild-type p53 has been shown to inhibit the activation of mTOR signaling via AMP-activated protein kinase (AMPK)/Tuberous sclerosis proteins 1 and 2 (TSC1/2), while mutant p53 enhances oxidative stress [47, 48]. These results indicate that mutations in *p53* and other loci may affect the MHY1485 sensitivity of cells. Furthermore, our study revealed several additional observations that will need to be addressed. First, our results showed that mitochondrial superoxide levels are increased in CT26 but decreased in LLC after irradiation (Fig. 4C). These opposing results may reflect dependence on mutations in various genes, including *p53* [49]. Second, we found that MHY1485 treatment had only limited effects on the cell cycle (Supplementary Data 5). However, the histograms that we obtained for the cell cycle were broad; presumably, the cell cycle of these treated cells should be reanalyzed using other methods such as BrdU assays. Third, MHY1485 treatment led to attenuation of *p21* mRNA levels in CT26 under irradiation conditions (Fig. 5B). It is possible that *p21* transcription is prevented by ER stress via induction of expression of the p53/47 isoform of p53 [50]. Fourth, MHY1485 treatment did not change p-mTOR levels, but decreased p-S6 and p-4E-BP1 levels in CT26 under non-irradiation condition (Fig. 2). It has been reported that p-S6 and p-4E-BP1 levels also are controlled by protein phosphatase 1 and the Mitogen-activated protein kinase kinase (MEK)/Extracellular signal-regulated kinases (ERK) pathway [51, 52]. Fifth, MHY1485 treatment decreased mitochondrial volume under non-irradiation conditions, but increased mitochondrial volume under irradiation conditions in both CT26 and LLC. Thus, MHY1485 may regulate the mitochondrial structure and dynamics. Sixth, we only analyzed apoptosis and senescence in the present study. Recently, however, several types of novel cell death, such as ferroptosis and necroptosis, have been reported [53, 54]. Further studies will be needed to investigate whether these other cell death pathways are induced by MHY1485 treatment.

Although our study did raise these additional questions, our take-home message is that *in vitro* treatment with MHY1485 increased radiosensitivity and decreased proliferation in both CT26 and LLC (Fig. 1); these effects were associated, at least in part, with increased apoptosis and senescence via ER stress overloading (Figs 3–5). These results suggest that MHY1485 is a promising radiosensitizing drug. Furthermore, other work has shown that MHY1485 may activate CD8+ T cells [12] and promote follicular helper T cell differentiation via activation of mTOR [55], suggesting that MHY1485 may provide positive effects in anti-cancer immunity [56]. Regarding the effects of MHY1485 treatment on normal tissue, MHY1485-induced mTOR activation may accelerate aging [57], and ER stress may reduce the normal tissue tolerance dose in radiotherapy. Future studies will be needed to investigate the detailed mechanisms of the combinatorial effects of MHY1485 and irradiation. Notably, our analysis will need to be extended to animal experiments.

## Supplementary Material

2020MHY-SpFig-sun4_rrab057
