# Supplementary material for: MHY1485 enhances X-irradiation-induced apoptosis and senescence in tumor cells
Source: J Radiat Res. 2021 Jul 16;62(5):782–92. doi: 10.1093/jrr/rrab057 (PMC8438247; doi:10.1093/jrr/rrab057)
Supplement: 2020MHY-SpFig-sun4_rrab057 [file 2020mhy-spfig-sun4_rrab057.pdf]

Supplementary Data 1

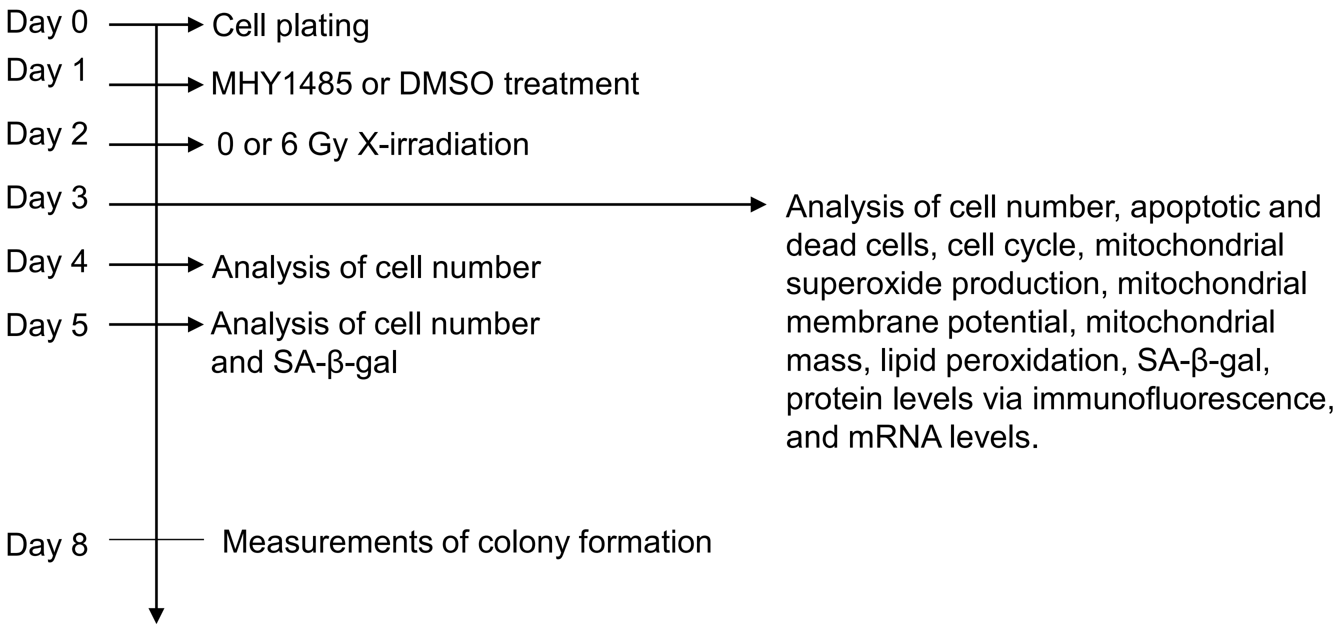

Supplementary Data 2

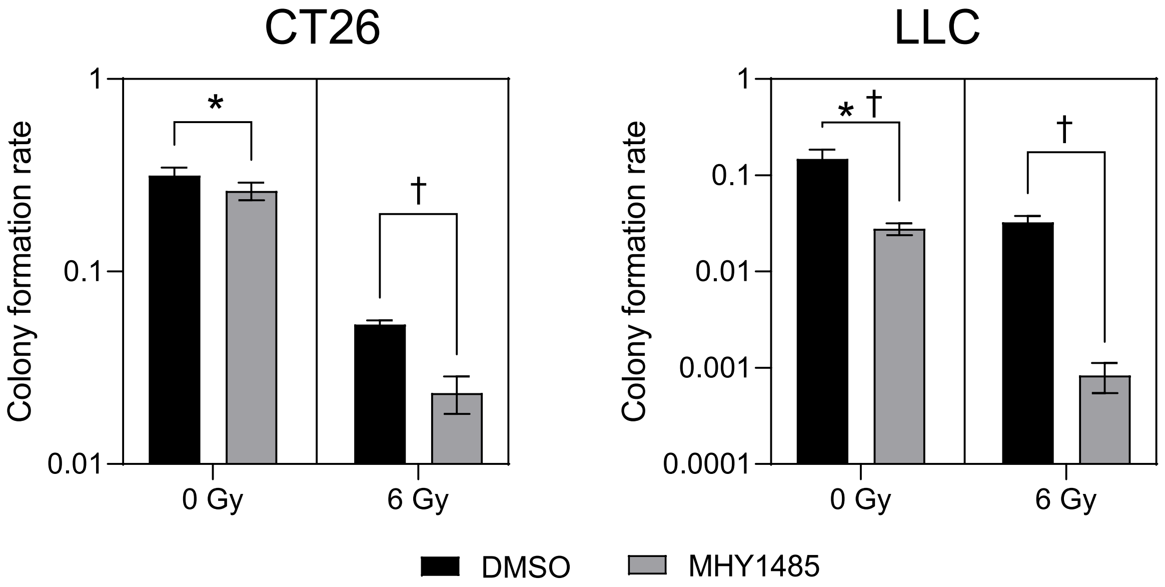

Supplementary Data 2. Colony formation rate. All quantitative data are presented as mean ± SD (n = 3). * p<0.05, two-way ANOVA with post hoc Sidak's multiple comparisons test vs. respective DMSO control. † p<0.05, two-tailed Student's t test vs. respective DMSO control.

Supplementary Data 3

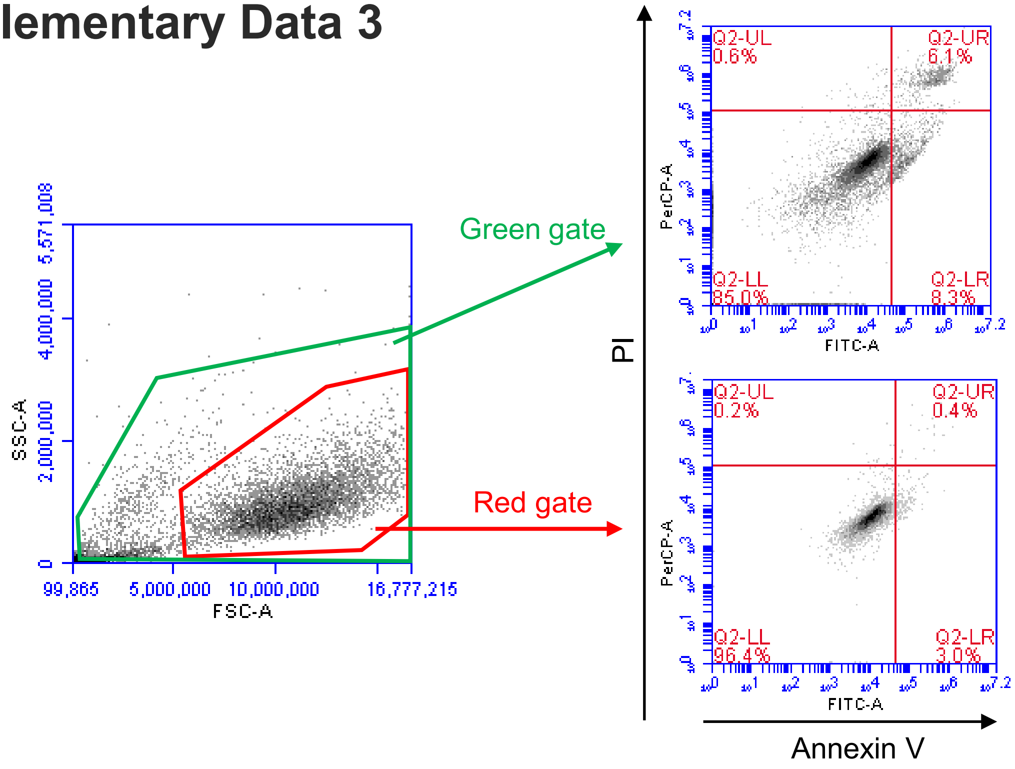

Supplementary Data 3. Gating of FSC-SSC panel in live cell staining. For apoptosis analysis (annexin V and JC-1), the green gate was set to include all cells. For senescence analysis (SPiDER-βGal, MitoBright, MitoSOX, and Liperfluo), the gate was used to excluding annexin V- and PI-positive cells.

Supplementary Data 4

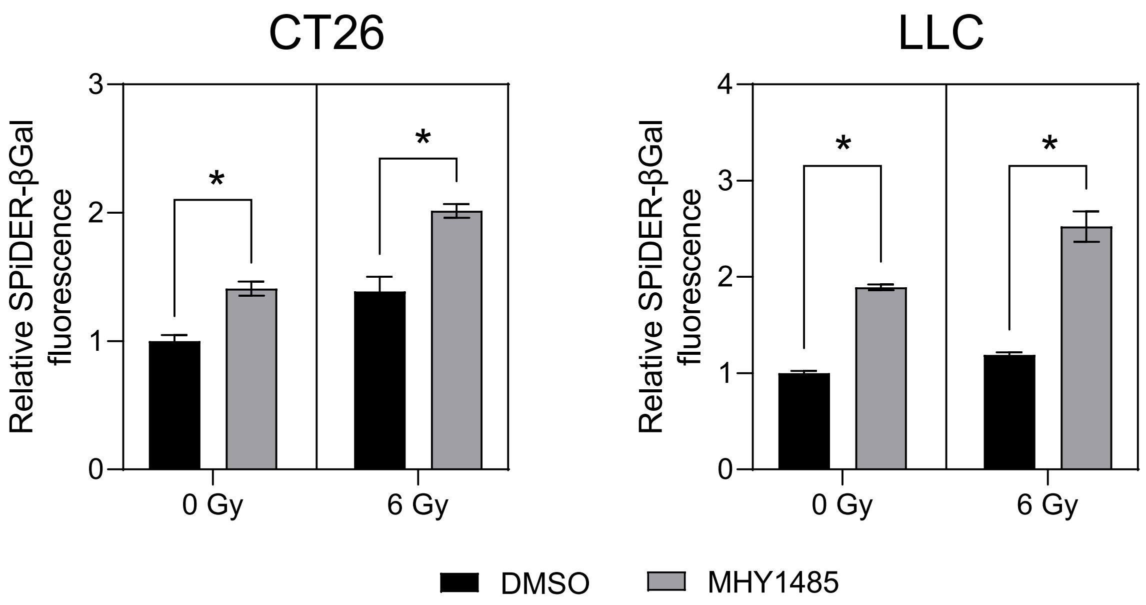

Supplementary Data 4. Relative senescence-associated β-galactosidase levels on Day 5. All quantitative data are presented as mean ± SD (n = 3). * p<0.05, two-way ANOVA with post hoc Sidak's multiple comparisons test vs. respective DMSO control.

Supplementary Data 5

Representative profiles of flow cytometry

(Red line indicates peak in 0 Gy + DMSO groups.)

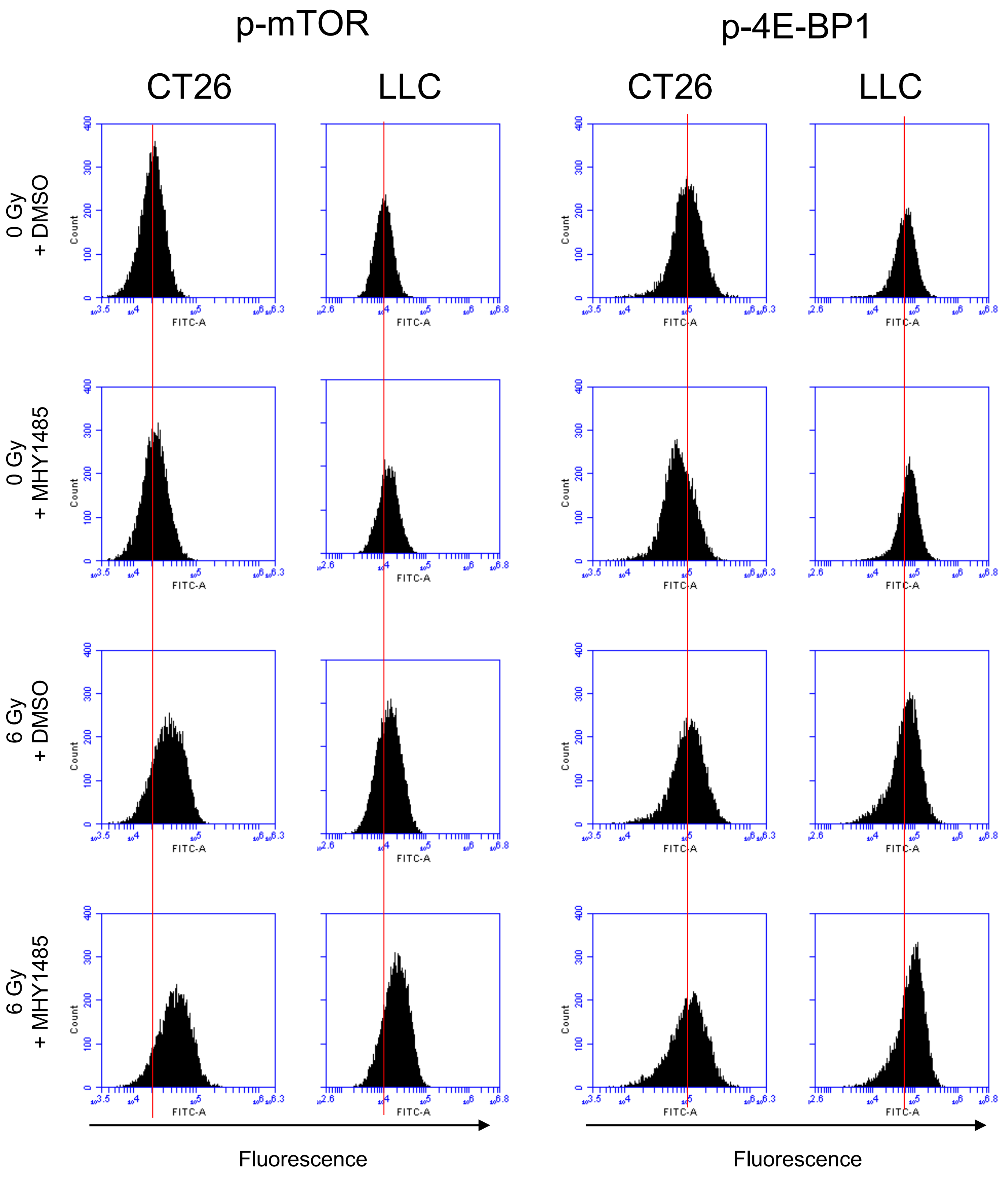

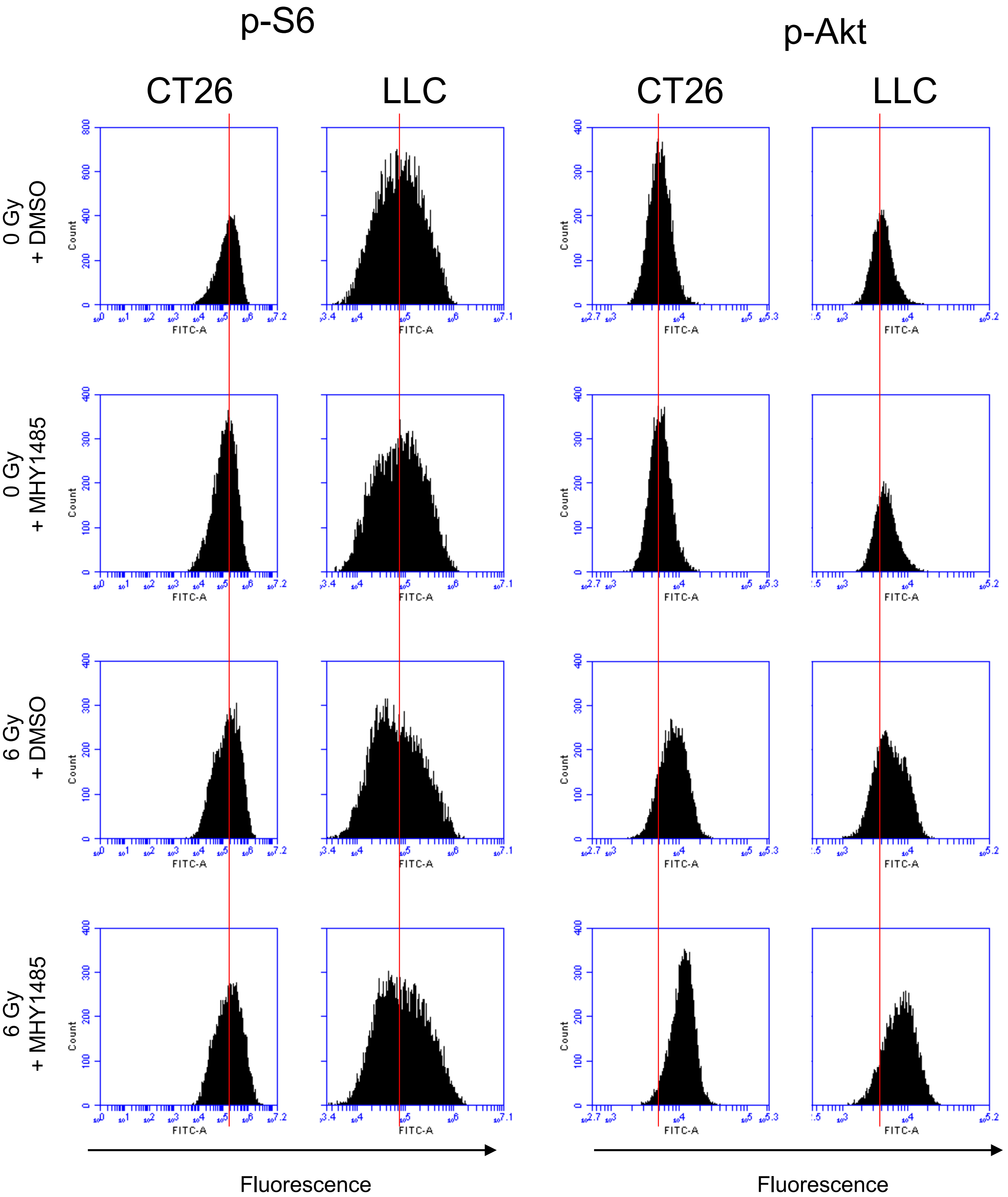
p-S6
p-Akt
CT26
LLC
CT26
LLC
0 Gy + DMSO
0 Gy + MHY1485
6 Gy + DMSO
6 Gy + MHY1485
Count
FITC-A
Fluorescence
Fluorescence

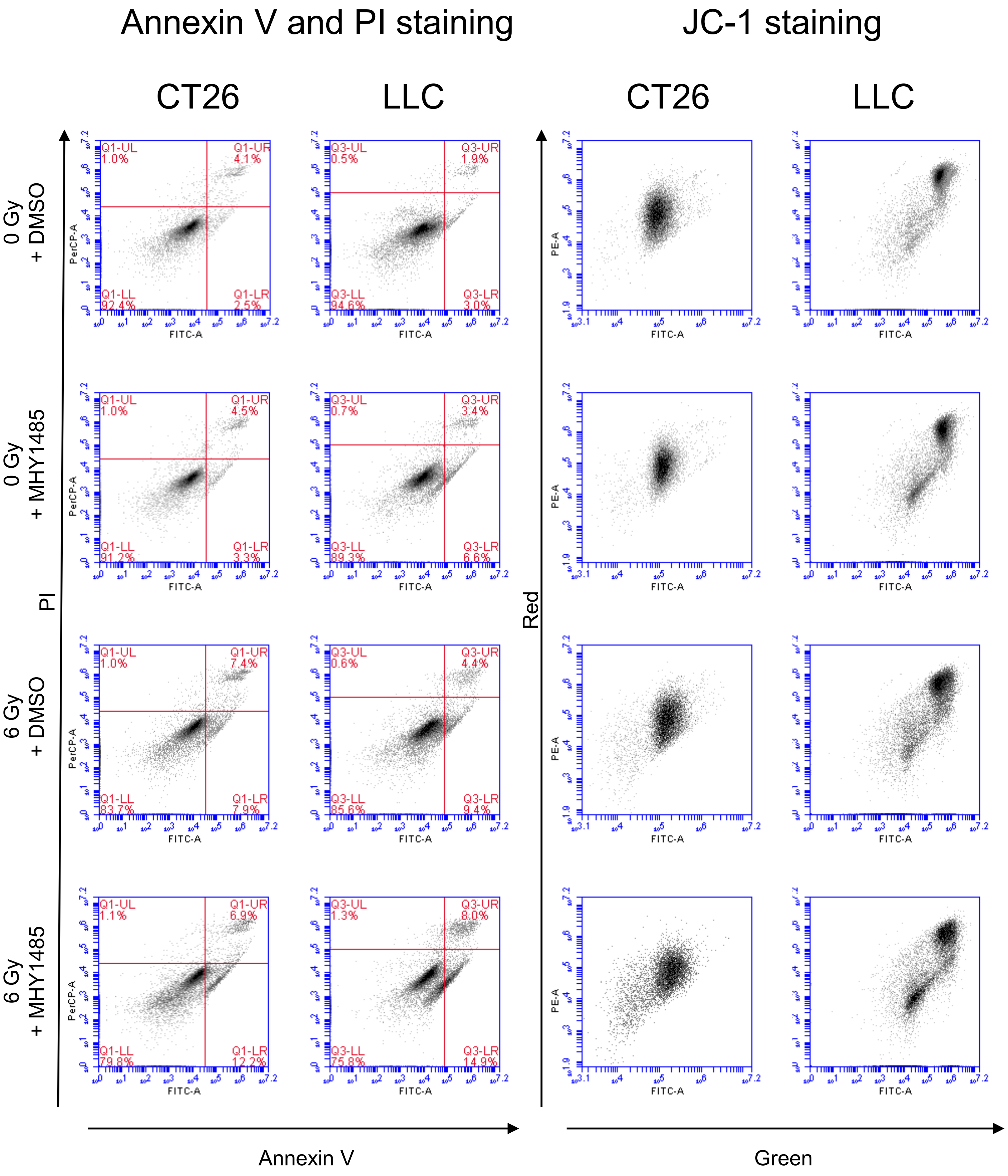
Annexin V and PI staining
JC-1 staining
CT26
LLC
CT26
LLC
0 Gy + DMSO
0 Gy + MHY1485
6 Gy + DMSO
6 Gy + MHY1485
PI
Red
Annexin V
Green
Q1-UL 1.0%
Q1-UR 4.1%
Q1-LL 92.4%
Q1-LR 2.5%
Q3-UL 0.5%
Q3-UR 1.9%
Q3-LL 94.6%
Q3-LR 3.0%
Q1-UL 1.0%
Q1-UR 4.5%
Q1-LL 91.2%
Q1-LR 3.3%
Q3-UL 0.7%
Q3-UR 3.4%
Q3-LL 89.3%
Q3-LR 6.6%
Q1-UL 1.0%
Q1-UR 7.4%
Q1-LL 83.7%
Q1-LR 7.9%
Q3-UL 0.6%
Q3-UR 4.4%
Q3-LL 85.6%
Q3-LR 9.4%
Q1-UL 1.1%
Q1-UR 6.9%
Q1-LL 79.8%
Q1-LR 12.2%
Q3-UL 1.3%
Q3-UR 8.0%
Q3-LL 75.8%
Q3-LR 14.9%
PerCP-A
FITC-A
PE-A

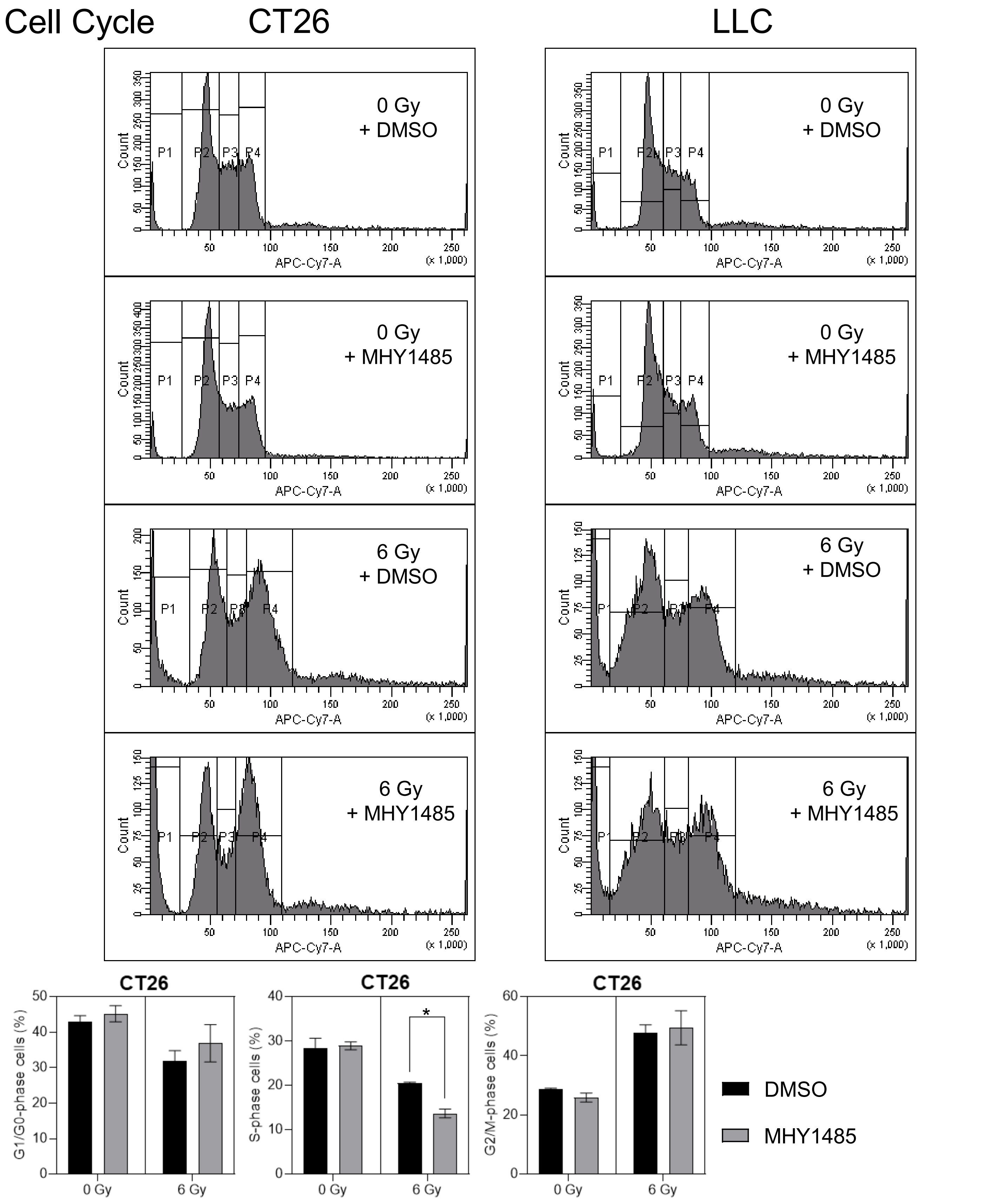

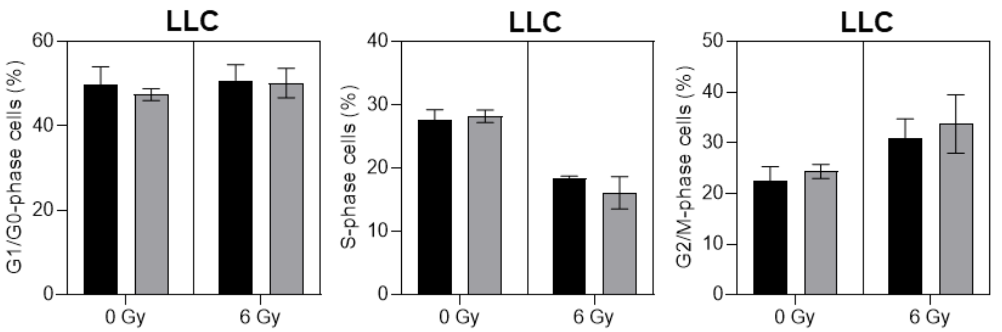

The quantitative data are presented as mean ± SD (n = 3). * $p<0.05$, two-way ANOVA with post hoc Sidak's multiple comparisons test vs. respective DMSO control.

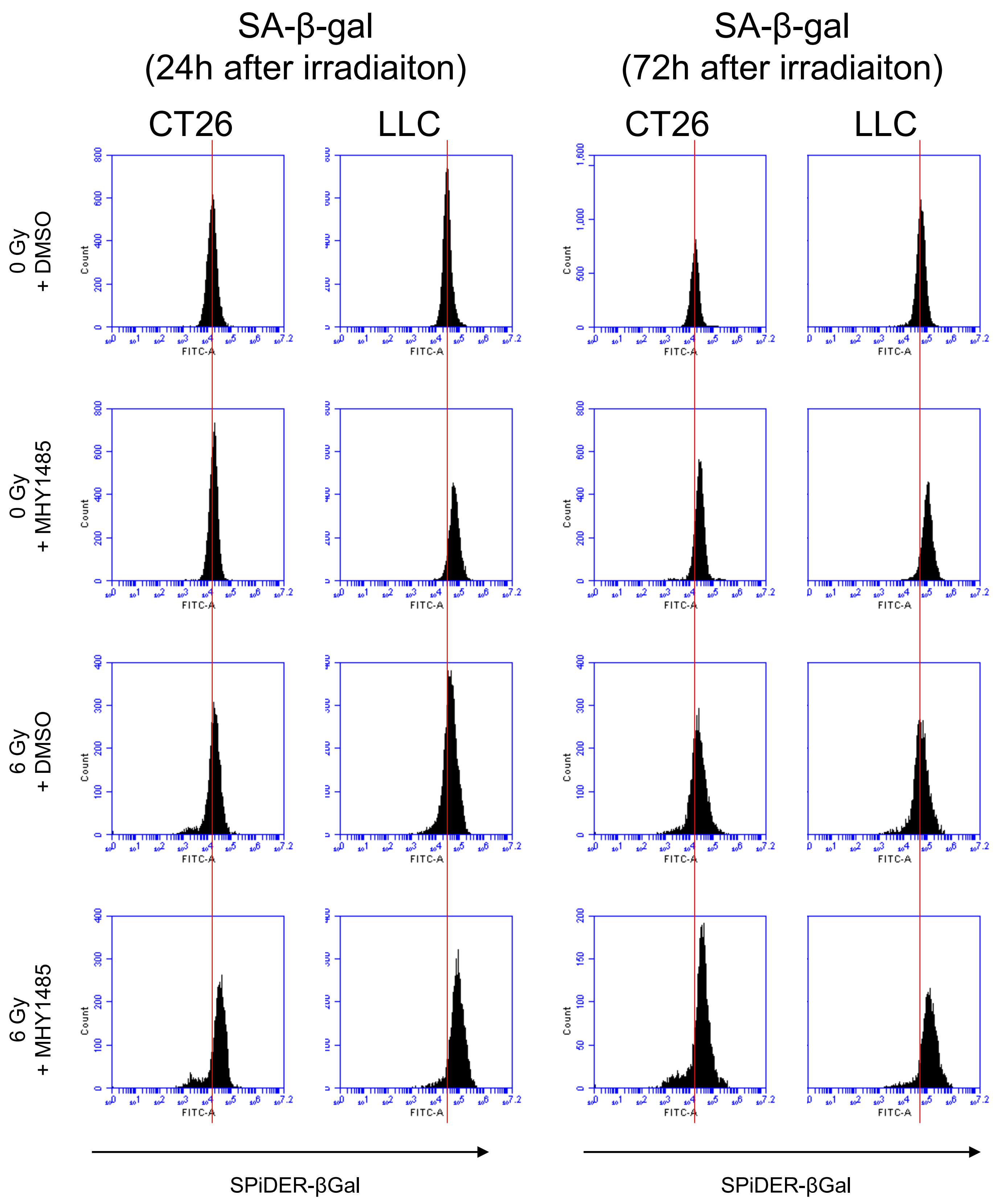
SA-β-gal
(24h after irradiaiton)
SA-β-gal
(72h after irradiaiton)
CT26
LLC
CT26
LLC
0 Gy
+ DMSO
0 Gy
+ MHY1485
6 Gy
+ DMSO
6 Gy
+ MHY1485
Count
FITC-A
SPiDER-βGal
SPiDER-βGal

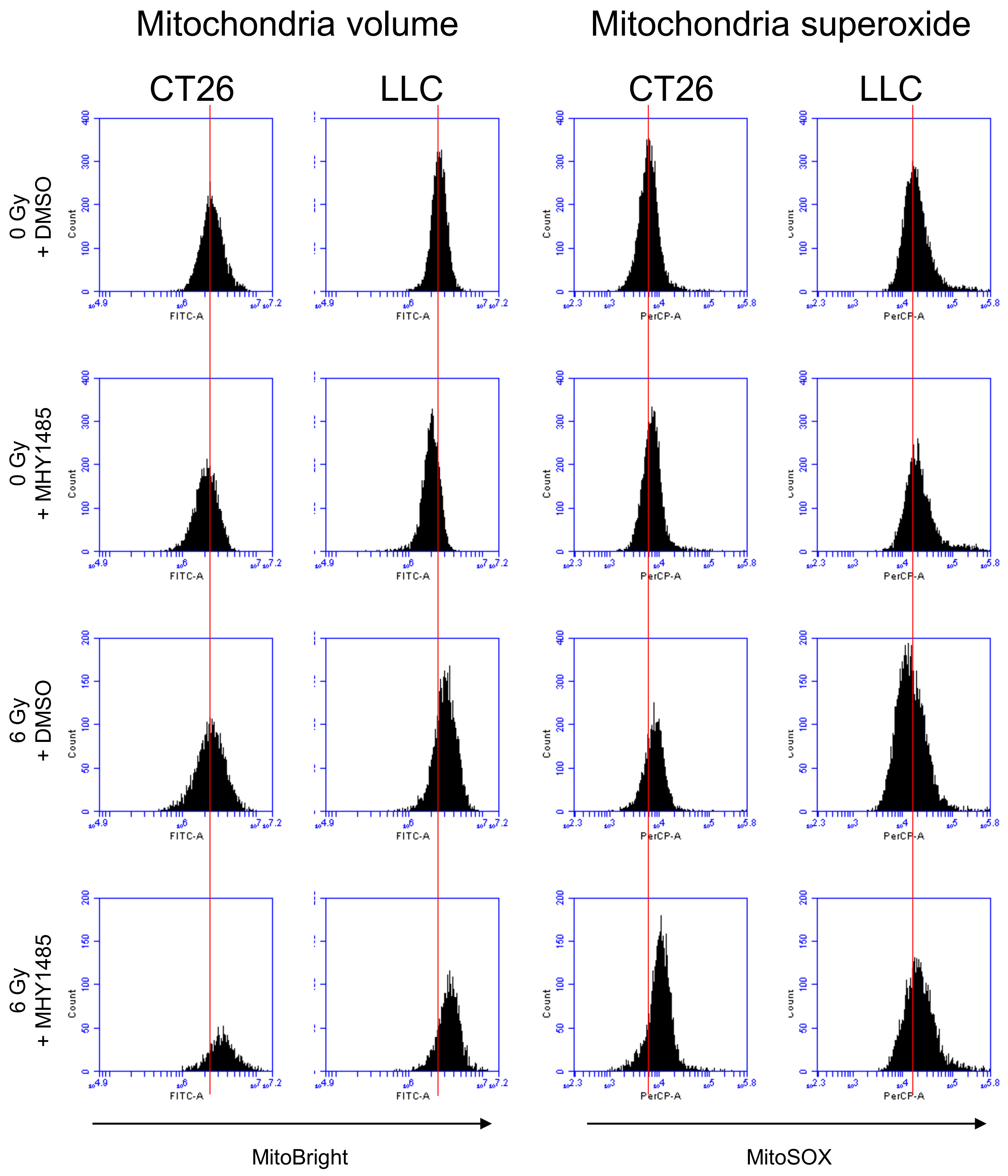
Mitochondria volume
Mitochondria superoxide
CT26
LLC
CT26
LLC
0 Gy + DMSO
0 Gy + MHY1485
6 Gy + DMSO
6 Gy + MHY1485
Count
FITC-A
PerCP-A
MitoBright
MitoSOX

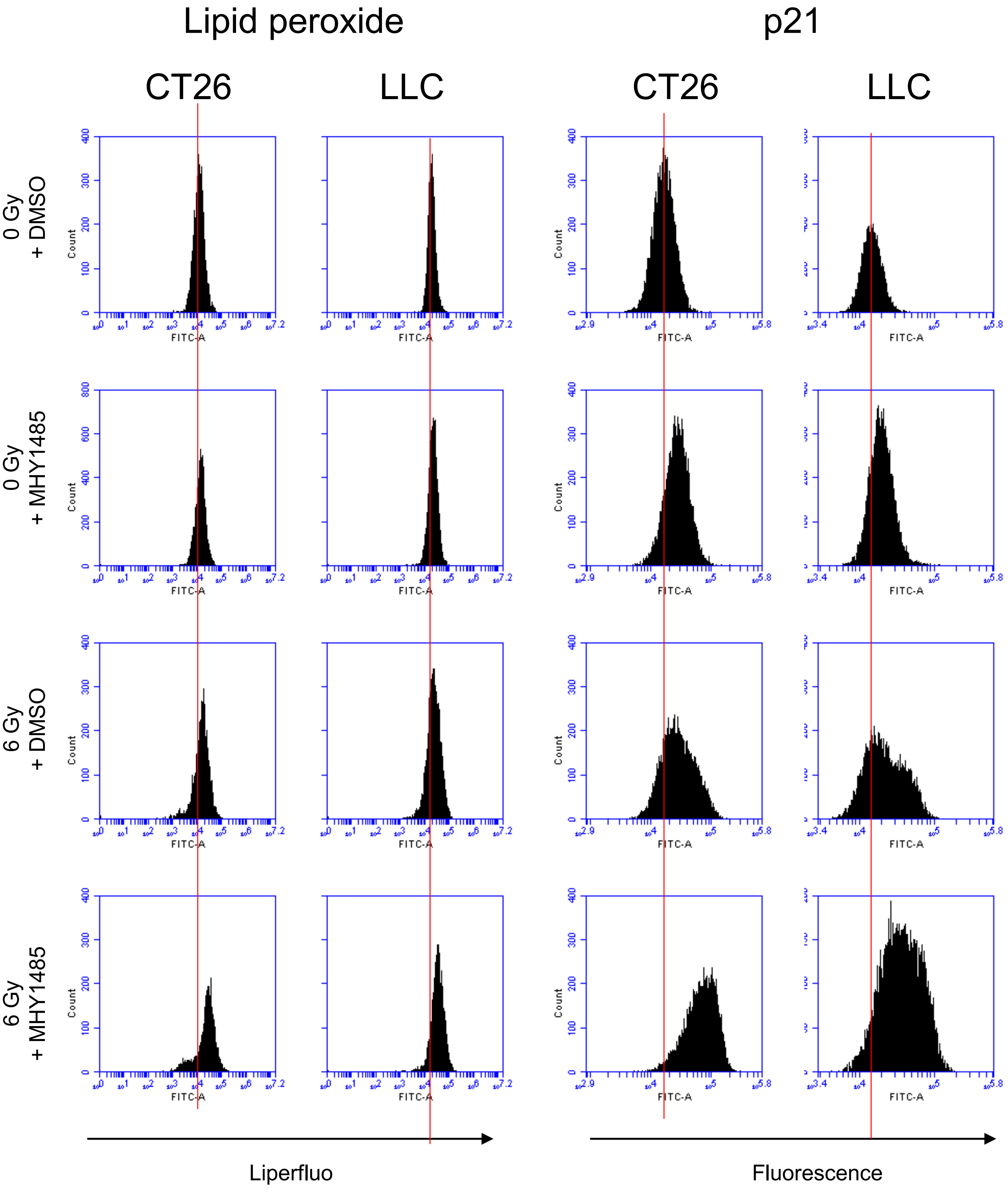

Lipid peroxide
p21
CT26
LLC
CT26
LLC
0 Gy + DMSO
0 Gy + MHY1485
6 Gy + DMSO
6 Gy + MHY1485
Count
FITC-A
Liperfluo
Fluorescence

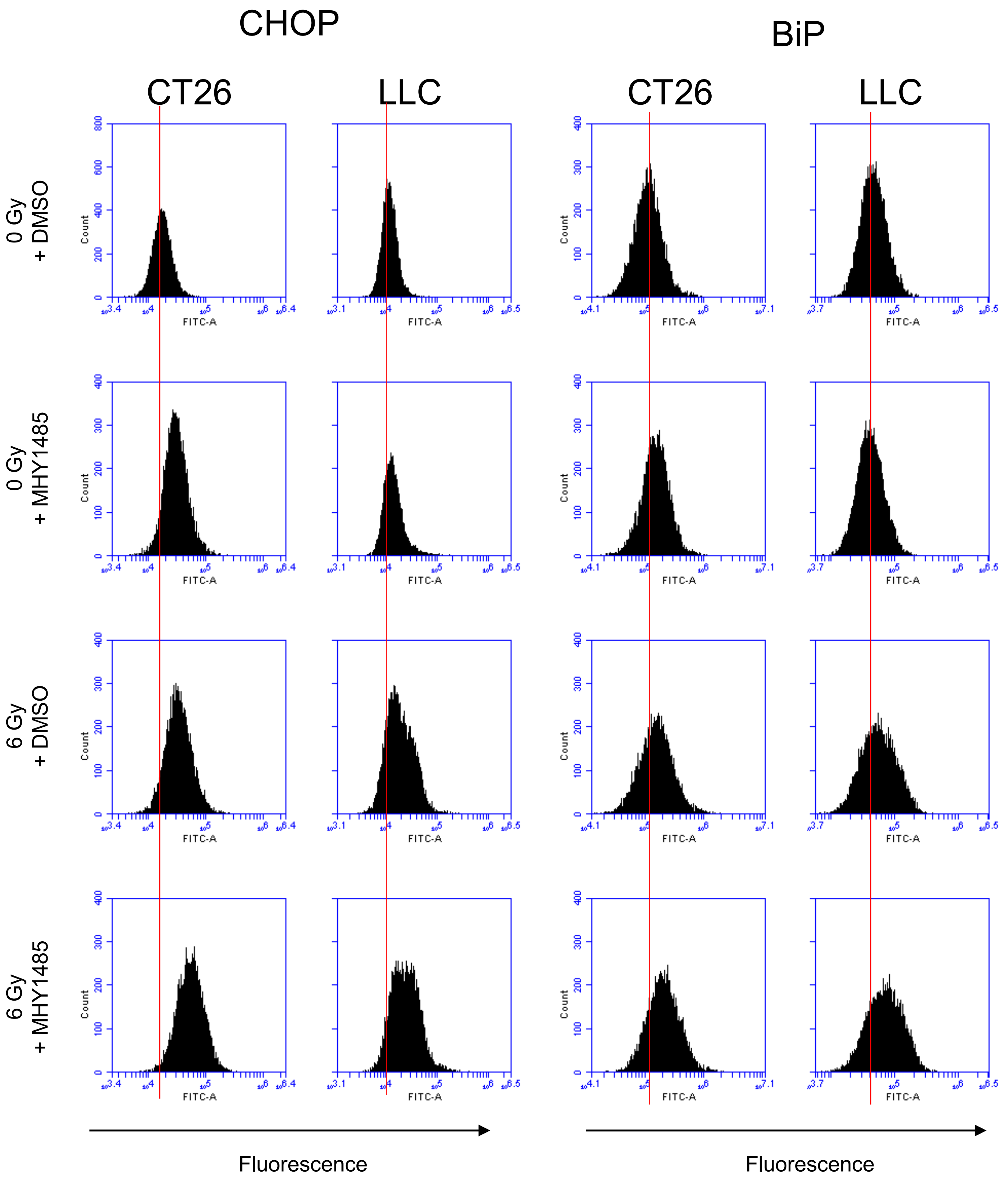

CHOP
BiP
CT26
LLC
CT26
LLC
0 Gy + DMSO
0 Gy + MHY1485
6 Gy + DMSO
6 Gy + MHY1485
Count
FITC-A
Fluorescence
Fluorescence

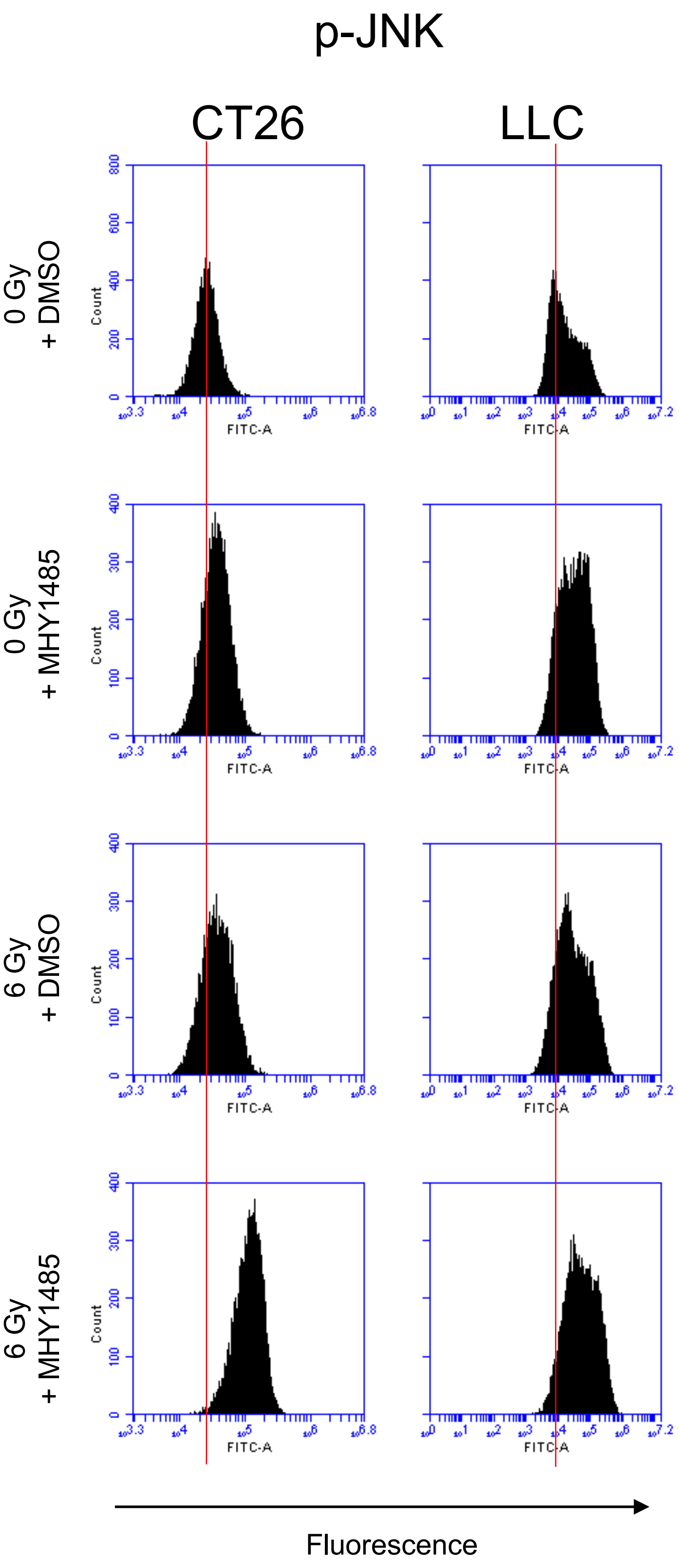
p-JNK
CT26
LLC
0 Gy
+ DMSO
0 Gy
+ MHY1485
6 Gy
+ DMSO
6 Gy
+ MHY1485
Count
FITC-A
Fluorescence
